# p15^PAF^ binding to PCNA modulates the DNA sliding surface

**DOI:** 10.1093/nar/gky723

**Published:** 2018-08-08

**Authors:** Matteo De March, Susana Barrera-Vilarmau, Emmanuele Crespan, Elisa Mentegari, Nekane Merino, Amaia Gonzalez-Magaña, Miguel Romano-Moreno, Giovanni Maga, Ramon Crehuet, Silvia Onesti, Francisco J Blanco, Alfredo De Biasio

**Affiliations:** 1 Structural Biology Laboratory, Elettra-Sincrotrone Trieste S.C.p.A., Trieste 34149, Italy; 2 Institute of Advanced Chemistry of Catalonia (IQAC), CSIC, Jordi Girona 18–26, 08034, Barcelona, Spain; 3 Institute of Molecular Genetics, IGM-CNR, via Abbiategrasso 207, 27100 Pavia, Italy; 4 CIC bioGUNE, Parque Tecnológico de Bizkaia Edificio 800, 48160 Derio, Spain; 5 IKERBASQUE, Basque Foundation for Science, Bilbao, Spain; 6 Leicester Institute of Structural & Chemical Biology and Department of Molecular & Cell Biology, University of Leicester, Lancaster Rd, Leicester LE1 7HB, UK

## Abstract

p15^PAF^ is an oncogenic intrinsically disordered protein that regulates DNA replication and lesion bypass by interacting with the human sliding clamp PCNA. In the absence of DNA, p15^PAF^ traverses the PCNA ring via an extended PIP-box that contacts the sliding surface. Here, we probed the atomic-scale structure of p15^PAF^–PCNA–DNA ternary complexes. Crystallography and MD simulations show that, when p15^PAF^ occupies two subunits of the PCNA homotrimer, DNA within the ring channel binds the unoccupied subunit. The structure of PCNA-bound p15^PAF^ in the absence and presence of DNA is invariant, and solution NMR confirms that DNA does not displace p15^PAF^ from the ring wall. Thus, p15^PAF^ reduces the available sliding surfaces of PCNA, and may function as a belt that fastens the DNA to the clamp during synthesis by the replicative polymerase (pol δ). This constraint, however, may need to be released for efficient DNA lesion bypass by the translesion synthesis polymerase (pol η). Accordingly, our biochemical data show that p15^PAF^ impairs primer synthesis by pol η–PCNA holoenzyme against both damaged and normal DNA templates. In light of our findings, we discuss the possible mechanistic roles of p15^PAF^ in DNA replication and suppression of DNA lesion bypass.

## INTRODUCTION

Sliding clamps are ring-shaped proteins that tether polymerases and other factors of the replisome to the genomic template, enabling DNA replication and repair. The molecular architecture of sliding clamps is conserved in all domains of life. Proliferating Cell Nuclear Antigen (PCNA)—the eukaryotic sliding clamp—is an 86-kDa homotrimeric ring with a six-fold pseudosymmetric rotation axis running through the centre of the clamp and a central channel lined with lysine and arginine-rich α-helices through which DNA is threaded (1–6). Experimental and computational analyses of the human PCNA–DNA complex showed that the DNA in the channel is tilted and its phosphates transiently interact with a set of basic residues forming a right-hand spiral that matches the DNA pitch (5,7). This dynamic interaction may allow the clamp to slide by rotationally tracking the DNA helix, or by a linear motion uncoupled from the helical pitch, or by a combination of the two modes (8). A recent report based on coarse-grained MD simulations (9) supports that the coupling between rotation and translation in PCNA sliding is weak, and that the translational motion is much faster than the rotational one, suggesting that PCNA slides on the double helix like a washer on a screw, rather than a nut on a screw. Importantly, growing evidence confirms the earlier observation that the PCNA–DNA interaction is critical for the function of the polymerase bound to the clamp (10). A recent report showed that acetylation of conserved lysine residues at the sliding surface of yeast PCNA is induced by DNA lesions and stimulates repair by homologous recombination (11). Acetylation of K20 negatively affects the processivity of the replicative polymerase δ (pol δ), but not that of the translesion synthesis (TLS) polymerase η (pol η), specialized in traversing DNA lesions such as thymine dimers or cisplatin adducts, suggesting that the modulation of the PCNA–DNA interaction can regulate the function of polymerases.

The front face of the PCNA ring contains the site of interaction of polymerases and other proteins, named the PIP-box binding site (3,12). The back face contains the sites of ubiquitylation and sumoylation (13,14). PIP-box interacting partners bind PCNA through a short consensus sequence with the pattern *QXXhXXaa*, where *h* is an aliphatic-hydrophobic residue, *a* aromatic-hydrophobic and *X* any residue (3,12).

The PCNA–associated factor p15^PAF^ (hereafter named p15) is an oncogenic, 11 kDa intrinsically disordered protein that regulates DNA replication and lesion bypass via a PIP-box interaction with PCNA (4,15–18). p15 co-localizes with PCNA in the nucleus of proliferating cells mainly in the S phase of the cell cycle (18–20). Depletion of p15 significantly decreases DNA synthesis (17–20), suggesting that p15 modulates the function of PCNA as a processivity factor. Co-immunoprecipitation from pancreatic cancer cell lines suggests that p15 is part of a DNA-replication complex with PCNA, pol δ and the endonuclease Fen-1 in replication foci (18). During unperturbed DNA replication, PCNA-bound p15 is mono-ubiquitylated at two N-terminal lysines (18) (K14 and K25). UV-induced replication stalling triggers the recruitment of pol η to the damaged site and the degradation of ubiquitylated p15 (18), and Povlsen and co-workers proposed that p15 may compete with pol η for binding to PCNA. However, the molecular mechanisms underlying the function of p15 in DNA replication and DNA lesion bypass remain unclear.

We have previously shown that, in absence of DNA, up to three p15 molecules bind the human trimeric PCNA ring at a site that extends from the PIP-box binding pocket to the clamp inner channel (4), and that the disordered p15 N-termini exit the clamp from the back face and directly interact with DNA (4,21). Negative stain electron microscopy of a p15–PCNA–DNA ternary complex showed particles with DNA in the clamp channel (4), but the molecular details of this assembly remained undefined.

In this work, we characterized the structure of ternary complexes composed of PCNA, DNA and the PCNA-interacting region of p15, by combining experimental and computational approaches. The co-crystal structure of PCNA in complex with two p15 molecules and a 10 bp primed DNA substrate, solved at 3.2 Å resolution, shows the duplex portion of DNA in the PCNA channel leaning towards the subunit not occupied by p15. The p15 residues N-terminal to the PIP-box contact four helices on the ring inner wall, partly shielding the DNA binding site of two subunits. This molecular arrangement is recapitulated by MD simulations of PCNA in complex with two p15 fragments with longer N-termini and a 40 bp DNA duplex. Solution NMR experiments show that DNA does not displace p15 from the inner rim of a PCNA ring in which the three subunits are occupied by p15. Accordingly, when PCNA is co-crystallized with three p15 peptides and DNA, the electron density map does not show features of DNA in the clamp channel. Thus, p15 outcompetes DNA for a common binding site in the clamp channel, and the stoichiometry of binding dictates the available sliding surfaces. Given the inhibitory activity of p15 in TLS (18), we hypothesized that the constraint imposed by p15 to DNA may need to be released for efficient DNA lesion bypass by the TLS polymerase pol η. This agrees with our data showing that p15 downregulates the activity of pol η–PCNA holoenzyme in bypassing a cisplatin-induced DNA lesion and in extending the corresponding undamaged template. Based on our findings, we discuss the possible mechanistic roles of p15 in DNA replication and lesion bypass.

## MATERIALS AND METHODS

### Protein expression and DNA duplexes

Human PCNA (UniProt: P12004) was produced in *Escherichia coli* BL21(DE3) cells grown in appropriate culture media to obtain protein with natural isotopic abundance or uniform enrichment using a clone with N-terminal His6-tag and PreScission protease cleavage site in a pET-derived plasmid. For NMR samples the protein was purified from the soluble fraction by Co^2+^-affinity chromatography, cleaved by PreScission protease and polished by gel filtration chromatography (22). All columns and chromatography systems used where from GE Healthcare. Protein elution was monitored by absorbance at 280 nm and confirmed by SDS-PAGE. The purified protein contained the extra sequence GPH- at the N-terminus. The PCNA sample for crystallization was obtained by introducing two additional purification steps (4). The sample cleaved with PreScission protease was dialyzed against 50 mM sodium acetate pH 5.5, 100 mM NaCl. After separation of some precipitated material, the solution was loaded on a HiTrap Heparin HP column equilibrated with the same buffer. After column washing, the protein was eluted with a 0–100% gradient of 50 mM sodium acetate pH 5.5, 2 M NaCl in 20 column volumes (CV). The protein containing fractions of the major peak were dialyzed against 20 mM Tris–HCl buffer pH 7.6, 150 mM NaCl and injected into a HiTrap Chelating HP column loaded with Co^2+^ cations to remove uncleaved PCNA. The flowthrough was loaded on a HiTrap Q Sepharose column and eluted with a 0–60% gradient of 20 mM Tris–HCl pH 7.6, 1 M NaCl in 5 CV. The protein containing fractions were concentrated and polished using a Superdex 200 26/60 column equilibrated with PBS, pH 7.0, and then exchanged into the crystallization buffer (20 mM Tris–HCl, pH 7.5, 10% glycerol, 2 mM DTT) using a PD10 column. Stock solutions in PBS or crystallization buffer were flash-frozen in liquid nitrogen and stored at −80°C. The protein concentrations were measured by absorbance at 280 nm using the extinction coefficient calculated from the amino acid composition (15 930 M^−1^ cm^−1^). All indicated concentrations of PCNA samples refer to protomer concentrations. dsDNA and pDNA duplexes were obtained by mixing equimolar amounts of the appropriate oligonucleotides, at 93°C for 2 min with subsequent annealing by slow cooling at room temperature.

### PCNA complexes crystallization and structure determination

#### p15^50–77^–PCNA–pDNA complex

Stocks of PCNA, p15^50–77^ and pDNA solutions were mixed to final concentrations of 0.4, 0.5 and 1.1 mM, respectively (1:1.2:2.7 protein monomer:peptide:pDNA molar ratio), and incubated at room temperature for 30 min before screening crystallization conditions using the hanging drop vapour diffusion method. Best diffracting co-crystals grew within 2 days at 4°C in droplets obtained by mixing 1 μl of the complex solution and 1 μl of a solution containing 10% polyethylene glycol 3350 in 0.1 M sodium acetate buffer, pH 4.5. The best crystals from the p15^50–77^–PCNA–DNA complex diffracted at 3.2 Å resolution on the ESRF-ID29 beamline, and belonged to *P*2_1_ space group. XDS (23) and the CCP4i suite (24) were used for data processing. Molecular replacement was used to place one hPCNA trimer (PDB ID: 4D2G) in the asymmetric unit after removing p15^50–77^ molecule and solvent. Several cycles of refinement using REFMAC5 (25) and model building using COOT (26) were carried out before placing the two p15^50–77^ chains into the *F*_o_ – *F*_c_ electron density map. NCS and TLS restraints were used. Inspection of the resulting unbiased difference Fourier's map inside the PCNA ring showed some electron density for one DNA strand of the double helix. Due to the disorder and/or partial occupancy the second DNA strand was only partially visible. However, modeling and refining the DNA in the same position as in the PCNA–DNA binary complex (5) gave rise to reasonable crystallographic parameters (i.e. model statistics, *B*-factor values and quality of the 2*F*_o_ – *F*_c_ map) and was consistent with the result from the MD simulations (see text for further details). Data collection and refinement statistics are listed in Supplementary Table ST2. Stereo view of 2*F*_o_ – *F*_c_ electron density map around the p15^50–77^ peptide with higher occupancy is displayed in Supplementary Figure S1. All figures with molecular models were prepared using PyMOL (www.pymol.org). Atomic coordinates and structure factors of p15^50–77^–PCNA–DNA complex have been deposited with PDB ID: 6EHT.

#### p15^41–72^–PCNA complex

Crystals of p15^41–72^–PCNA complex were obtained by hanging-drop vapor diffusion method at 4°C. Cubic crystals were grown on precipitant solution containing 28% polyethylene glycol 400, 0.2 M CaCl_2_ in 0.1 M Hepes pH 7.0 buffer. Stocks of PCNA, p15^41–72^ and DNA solutions were mixed to final concentrations of 0.5, 0.5 and 0.5 mM, respectively (1:1:1 protein monomer: peptide: DNA duplex molar ratio), and incubated at room temperature for 30 min before crystallization. Best diffracting co-crystals grew within 1 day and were flash-frozen directly, and diffracted to 2.9 Å resolution on the Elettra XRD1 beamline, and belonged to P2_1_ space group (Supplementary Table ST2). XDS (23) and the CCP4i suite (24) were used for data processing. Molecular replacement was used to place one hPCNA trimer (PDB ID: 1VYM) in the asymmetric unit. Several cycles of refinement using REFMAC5 (25) and model building using COOT (26) were carried out before placing the three p15^41–72^ chains into the *F*_o_ – *F*_c_ electron density map. Jelly-Body restraints were used. Inspection of the resulting unbiased difference Fourier's showed no electron density attributable to DNA inside the PCNA ring.

### MD simulations

Two 300 ns MD simulation replicas were performed for the same system. The system is a ternary complex composed of PCNA, two p15 peptides spanning residues 47–70 and a 40 bp DNA. The initial MD model was built by combining two different crystal structures: the p15^50–77^–PCNA complex (4) and the PCNA–dsDNA complex (5). The p15 peptide was designed based on the p15^50–77^–PCNA complex structure (4). The seven C-terminal residues are flexible and do not interact with PCNA and were thus excluded, while five extra residues at the flexible N-terminus were added as they are located in the clamp channel, and may transiently interact with DNA, resulting in a final segment p15^47–70^. The DNA segment, with 10 bp in the crystallographic structure, was also extended by 15 bp in each direction of the helical axis. Extension of all fragments was performed using COOT (26). The system was completed by adding TIP3P water molecules in a truncated dodecahedron box at least 1.5 nm away from the DNA or protein atoms. Cl- and Na+ ions were added for charge neutralization and to mimic experimental conditions of 100 mM salt concentration. The system was minimized and equilibrated for 0.1 ns in the NVT ensemble and then for 0.1 ns in the NPT ensemble. All calculations were performed using Gromacs 5.1 (27) and parmBSC1 force field (28), the trajectories were analyzed using MDTraj package (29) and plots were generated using Matplotlib Python Library (30). The stability of the simulations was checked by visual inspection of the trajectories and the RMSD with respect to the starting structure as plotted in Supplementary Figure S2. To track the evolution of the DNA position inside the PCNA ring (Figure 2B), we have followed this procedure: First, the PCNA chains are superimposed for all frames, to eliminate global rotation and translation. Second the centre of each PCNA chain is calculated for all trajectory frames. Third, DNA base-pairs 17–21 for chain F and 20–24 for chain G are selected as those being inside the PCNA ring. Its centre is also calculated for each frame. Fourth, to project into a 2D space, a Principal Component Analysis is performed for the PCNA chain centres, and DNA centres are projected into the first two components of this subspace.

### NMR spectroscopy

^1^H–^15^N TROSY spectra were recorded at 35°C on a Bruker Avance III 800 MHz (18.8 T) spectrometer equipped with a cryogenically cooled triple resonance z-gradient probe. A 400 ul sample of 100 μM U-[^2^H,^13^C, ^15^N,] PCNA in 20 mM sodium phosphate buffer, 50 mM NaCl, pH 7.0, 20 μM 2,2-dimethyl-2-silapentane-5-sulfonate, 0.01% NaN3, 1 mM DTT and 5% ^2^H_2_O was placed in a 5 mm Shigemi NMR tube (without plunger) and increasing volumes of p15^50–77^ or dsDNA stock solutions were added and mixed (by capping and inverting the tube). The peptide and the DNA stocks solutions were prepared in the same buffer as the PCNA samples (except that no NaN_3_, DSS or ^2^H_2_O was added). For that purpose, and to remove unwanted salts from the synthetic peptide and oligonucleotides, they were dissolved in 20 mM sodium phosphate buffer, 50 mM NaCl, pH 7.0 and desalted on a PD-10 Minitrap G25 column. For duplex formation, equimolar amounts were mixed and annealed (2 min at 95°C in a thermoblock followed by slow cooling down to room temperature). The duplex and the peptide were concentrated by ultrafiltration up to 20.84 mM (dsDNA) or 9.52 mM (p15^50–77^) and concentrated DTT was added up to 1 mM. Small volumes of the stock peptide solution were added stepwise to the PCNA samples, causing a 7% PCNA dilution. TROSY spectra were measured with 144 or 256 indirect points (alternating between 8 and 14 h total duration). The PCNA–p15^50–77^ sample remained clear during the 6-day long titration. When the peptide was present at an excess molar ratio of 6.4, the observed changes in the PCNA spectrum were judged to be within the experimental error with respect to the previous addition, and PCNA was considered to be saturated with the peptide. Then a volume of dsDNA stock was added to a 1:3 molar ratio (PCNA trimer:DNA duplex). Further additions of DNA did not cause further changes in the PCNA signals. The structural integrity of the DNA duplex was assessed from the imino signals observed in one-dimensional proton spectra, whose intensities increased upon duplex addition. The pH of the PCNA samples was measured at the beginning and at the end of the titrations inside the NMR tubes and found to deviate by less than 0.1 units. Therefore, the small measured shifts are not caused by differences in pH. The titration with the peptide allowed for an extensive transfer of NMR signal assignments from the free PCNA to the p15^50–77^-bound PCNA spectra (with a coverage of 72% of non proline residues). For the p15^50–77^-DNA-bound PCNA the assignment transfer covered 69% of the PCNA signals. The CSP caused by the peptide or the dsDNA were computed as the weighted average distance between the backbone amide ^1^H and ^15^N chemical shifts in the free and bound states (31,32).

### DNA synthesis assays

#### Chemicals

Deoxynucleotides were purchased from GeneSpin (Milan, Italy).

#### Oligonucleotides

The 24-mer template oligonucleotide containing the cis-PtGG adduct was a kind gift from S.J. Sturla (ETH, Zürich) and was prepared and purified as described previously (Nilforoushan, 2015). All other DNA oligonucleotides, all HPLC purified, were synthesized by Biomers.net (Germany). The 18mer primer oligonucleotide was 5′-labeled with carboxyfluorescin (FAM) group. The labeled primer was mixed to the complementary template oligonucleotide at 1:1 (M/M) ratio in the presence of 150 mM Hepes–KOH pH 7.4, 500 mM KCl, 10 mM MgCl_2_, 250 mM NH_4_Ac, heated at 95°C for 5 min and then slowly cooled at room temperature.

#### Human recombinant pol η

pJM879 (33), expressing N-terminal His-tagged human pol η, was a kind gift from R. Woodgate (NIH, USA). Human recombinant pol η was expressed and purified with a modified protocol: BL21 DE3 competent *E. coli* cells were transformed with pJM879. Plates containing 30 mg/ml kanamycin were used to identify kanamycin-resistant colonies that were picked and grown overnight at 37°C. DNA purified from bacterial cultures using NucleoSpin^®^ Plasmid (NoLid) kit (MACHEREY-NAGEL, Düren, Germany) was digested to confirm the presence of the gene expressing pol η. Transformed *E. coli* cells were used to inoculate a 50 ml starter culture. After overnight growth at 37°C, 12 ml of the starter culture were put into 1 l of LB medium (containing 30 mg/ml kanamycin). After 6 h growth at 37°C, cells were harvested by centrifugation and pellet frozen at –80°C. The pellet was resuspended in 40ml of lysis buffer (50mM Tris–HCl pH 7.5, 0.3 M NaCl, 20 mM imidazole, 10% glycerol, 10 mM b-mercaptoethanol [BME], 1× lysozime (Eurobio, Courtaboeuf, France), 1× ethylenediaminetetraacetic acid [EDTA]-free protease inhibitor (SIGMAFAST™ Protease Inhibitor Cocktail Tablets, Sigma-Aldrich), 1 mM phenylmethane sulfonyl fluoride [PMSF] and lysed through sonication. After ultracentrifugation at 98 000g at 4°C for 1.5 h, the supernatant was loaded onto a 1-ml Ni-NTA column (HisTrap™ HP, GE Healthcare). Column washing was performed with 3 ml of W1 buffer (20 ml Tris–HCl pH 7.5, 1 M NaCl, 20 mM imidazole, 10% glycerol, 10 mM BME) followed by 3 ml of W2 buffer (10 mM Na-phosphate pH 7.7, 0.3 M NaCl, 20 mM imidazole, 10% glycerol, 10 mM BME). Elution of target protein was obtained using buffer H (10 mM Na-phosphate pH 7.7, 0.3 M NaCl, 200 mM imidazole, 10% glycerol, 10 mM BME). The pol η positive fractions (0.5 ml each) were pooled and overnight dialyzed with buffer M (20 mM Na-phosphate pH 7.3, 0.1 M NaCl, 10% glycerol, 10 mM BME), in dialysis cassette (Slide-A-Lyzer™ Dialysis Cassettes, 3.5K MWCO, Thermo Scientific). Pool was then loaded onto a 1-ml cation exchanger column (HiTrap SP, Pharmacia Biotech). Buffer N (20 mM Na-phosphate pH 7.3, 0.5 M NaCl, 10% glycerol, 10 mM BME) was used to elute pol η with a linear gradient. The pol ηpositive fractions (0.5 ml each) were aliquoted and stored at –80°C in 20% final glycerol.

Recombinant ScRF-C was obtained as a kind gift from the laboratory of Alessandro Costa (Francis Crick Institute), and was prepared following the procedure described in (34).

#### Enzymatic assays

All reactions were performed in a 10 μl final volume using the following conditions: 40 mM Tris pH 8, 1 mM dithiotreitol (DTT), 0.25 mg/ml bovine serum albumin (BSA), 10 mM Mg^2+^ (unless otherwise stated in the figures or figure legends). Enzymes and DNA substrates concentrations are indicated in figure legends. Reactions were incubated at 37°C for 15 min, unless otherwise stated. Reaction mixtures were stopped by addition of standard denaturing gel loading buffer (95% formamide, 10 m methylenediaminetetraacetic acid, xylene cyanol and bromophenol blue), heated at 95°C for 5 min and loaded on a 7-M urea 12% polyacrylamide (PA) gel.

#### Electronic image manipulation

Linear transformations have been applied in some instance to the whole images using the exposure/brightness filters of Adobe Photoshop CS6 with the sole purpose of reducing excessive background. No masking/enhancement was applied to any specific feature of the images.

## RESULTS

### Crystallographic evidence for p15–PCNA–DNA interactions

**Figure 1.**
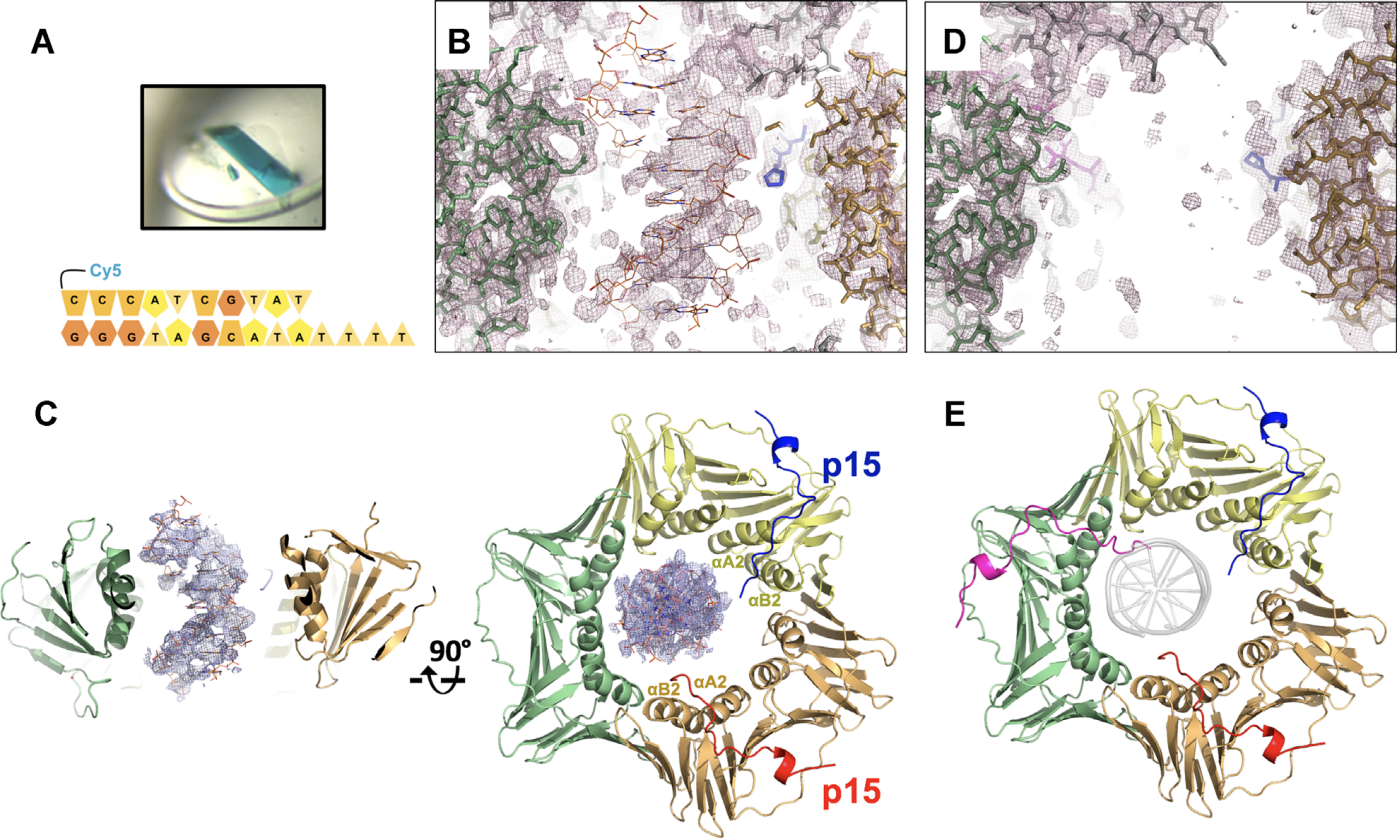
Crystal structures of human PCNA bound to p15 fragments and DNA. (**A**) Blue crystals of p15^50–77^–PCNA–DNA complex. Co-crystals of PCNA mixed with p15^41–72^ and DNA were also blue, confirming incorporation of DNA in the crystal lattice. The cartoon below shows the sequence of the DNA substrate. (**B**) Side view of the 2*F*_o_ – *F*_c_ omit map of the p15^50–77^–PCNA–DNA complex refined without DNA in the model, contoured at 0.7 σ, showing the PCNA central channel. PCNA subunits (green and wheat) and p15^50–77^ peptide in the background (blue) are in stick representation. The loop of a symmetry related PCNA molecule is shown in grey. The DNA, modeled as in the PCNA–dsDNA binary structure (5), is shown in orange. (**C**) Side- and top views of the refined p15^50–77^–PCNA–DNA complex structure. PCNA and p15^50–77^ are shown in ribbon representation, and the protein and peptide chains colored differently. The DNA, shown in orange, is modeled as in the PCNA–dsDNA binary structure. The 2*F*_o_ – *F*_c_ map around DNA is shown contoured at 0.7σ. (**D**) Side view of the 2*F*_o_ – *F*_c_ map of the p15^41–72^–PCNA complex contoured at 0.7σ, showing the PCNA central channel as in (B). (**E**) Top view of p15^41–72^–PCNA complex structure, color-coded as in (C). The DNA shown as a grey transparent ribbon in the same position as in (C) would cause a steric clash with the N-terminus of the p15 peptide on the third PCNA subunit.

We co-crystallized human PCNA with a 10-bp primed DNA duplex (pDNA, as seen in published crystallographic analysis with both bacterial clamp (35) and *Saccharomyces cerevisiae* PCNA (2) and either the p15 fragment that was previously co-crystallized with PCNA alone (p15^50–77^, comprising the extended PIP-box) (4), or a longer fragment that includes nine additional N-terminal residues (p15^41–72^). In both crystals, incorporation of DNA is confirmed by their blue color, due to the presence of a Cy5 probe attached to the DNA (Figure 1A).

Crystals including the p15^50–77^ peptide diffracted to 3.2 Å resolution, and the Fourier difference map calculated after placing and refining the PCNA ring alone in the asymmetric unit showed two PIP-box sites occupied by the p15^50–77^ peptide and electron density features in the channel that may be attributed to one strand of the DNA duplex. However, electron density from the second strand is weak and the 4-base single stranded overhang invisible (Figure 1B). This suggests partial disorder and/or low occupancy of DNA. We then aligned the previously determined structure of PCNA bound to a 10 bp dsDNA (5) onto the current model and found that the DNA duplex fits reasonably well the residual electron density in the ring channel (Figure 1B). This DNA position, which leans towards the PCNA subunit not occupied by p15^50–77^, also results in the best model statistics, lowest *B*-factors of DNA and best quality of the 2*F*_o_ – *F*_c_ electron density map (Figure 1C, and Supplementary Table ST2) and, importantly, agrees with the results from MD simulations (see below). The electron density for the DNA is still weak, indicating low occupancy and/or the presence of multiple conformations, but allows to propose a model, which shows the duplex portion of the DNA substrate in a similar position to that seen in the PCNA–dsDNA binary model, in the presence of two p15 peptides (Figure 1C). As in the p15^50–77^–PCNA binary structure (4), the stoichiometry of p15^50–77^ binding to PCNA is defined by the crystal packing, where a symmetry-related PCNA molecule occludes the peptide binding site on one subunit. The two p15^50–77^ peptides show different occupancies. The peptide with higher occupancy has its PIP-box (Q62-F69) sitting on at the PCNA front face forming a 3_10_ helical turn, with a type-I β-turn at its N-terminus (P59-Q62) that positions residues P52-T58 to contact PCNA helices αA2 and αB2 on the clamp inner wall (Figure 1C and Supplementary Figure S1). Features of the peptide with lower occupancy are comparable yet weaker, and the peptide N-terminus could be modelled up to V53. Because of the partial disorder of DNA and the presence of a symmetry related loop that plugs the top of the PCNA channel (and may potentially affect both DNA positioning and occupancy, Figure 1B), we resorted to MD simulations to corroborate the crystallographic results and gain further structural insights on the ternary assembly.

Co-crystals of PCNA with p15^41–72^ and pDNA were also obtained and diffracted to 2.9 Å resolution (Supplementary Table ST2). In the electron density map, however, no significant density was observed that may arise from DNA (Figure 1D), suggesting that, although incorporated in the crystal, the DNA is not sufficiently ordered to generate a structured signal. The electron density map showed three p15 fragments spanning residues 50–72 in the corresponding PIP-box sites, with a conformation analogous to that observed in the co-structure with the p15^50–77^ peptide (Figure 1E and Supplementary Figure S1). Notably, due to steric hindrance, the location of the p15^41–72^ peptides would interfere with DNA binding to PCNA in the orientation observed in the PCNA–dsDNA binary structure (Figure 1E), suggesting that p15 may outcompete DNA for binding to the PCNA inner rim.

### MD simulation of PCNA in complex with two p15 PIP-boxes and a 40 bp DNA

**Figure 2.**
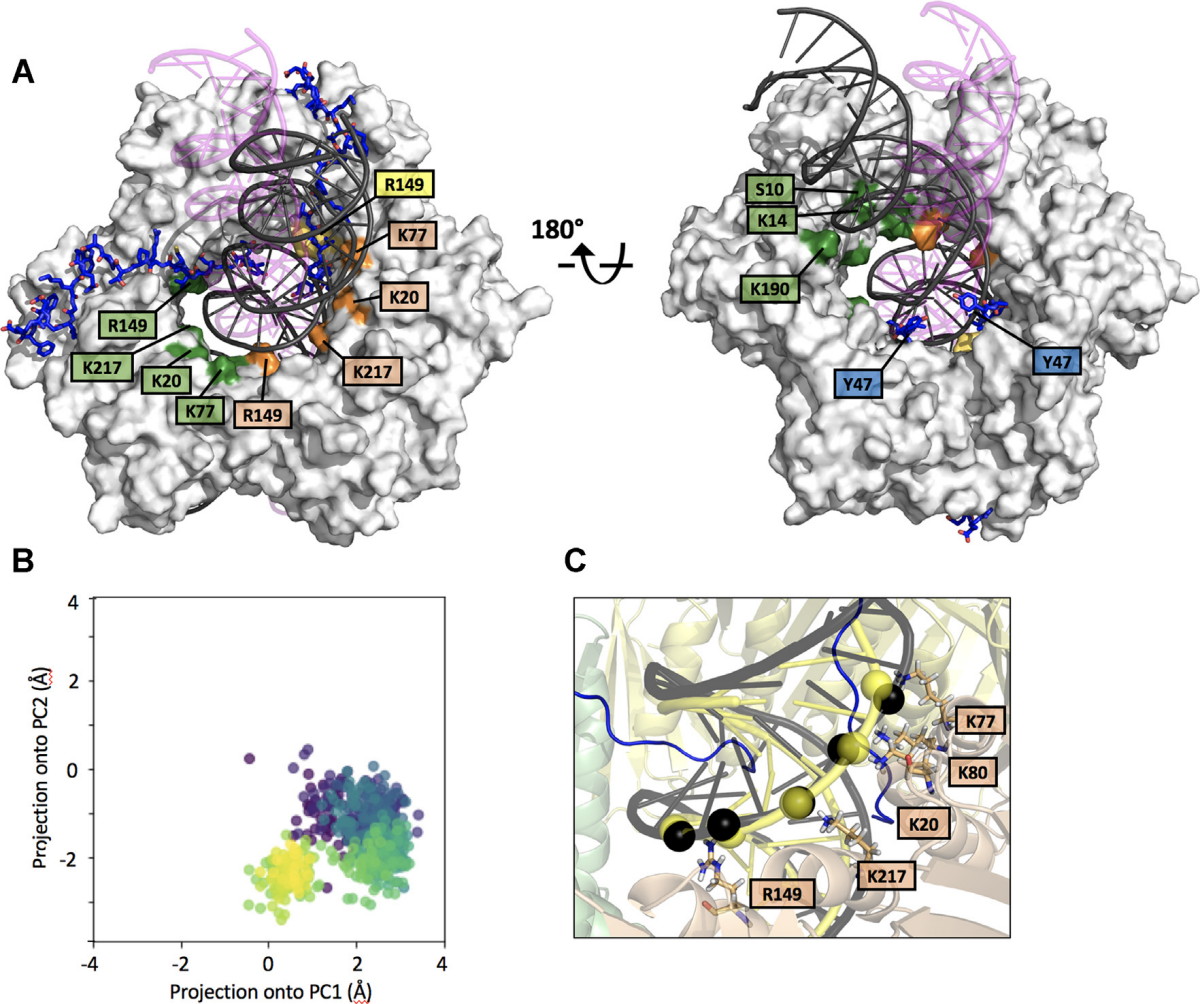
MD simulation of PCNA bound to two p15^47–70^ peptides and a 40 bp DNA (**A**) Superposition of the initial and equilibrium states of the MD trajectory. PCNA is shown as a gray surface and DNA as a ribbon. The DNA in magenta (with transparency), and black correspond to the initial and equilibrium states of the simulation, respectively. PCNA residues whose side chains are engaged in polar contacts with DNA phosphates are labeled. Residues of different PCNA subunits are colored in green, yellow and wheat. (**B**) Principal Component Analysis of the evolution of the DNA position inside the PCNA ring (see Methods section for details). The centre of DNA in each trajectory frame was projected onto the first 2 components of the subspace composed of the centres of the 3 PCNA subunits. Each frame is coloured using the viridis colormap, which goes from dark purple for the first frames to yellow for the last ones. In the initial frame, DNA is close to (0,0), the centre of the three PCNA chains, and it quickly translates to a non-centered position. The final position is retained due to the stabilizing interactions reported in Supplementary Figure S4. (**C**) Close-up of the equilibrium state of the MD trajectory showing the PCNA–DNA interface. Interacting PCNA side chains and DNA phosphates (interatomic side chain nitrogen – DNA phosphorus distance < 4 Å) are shown as sticks and black spheres, respectively. DNA in yellow corresponds to the position in the crystallographic PCNA–dsDNA binary structure (5), with interfacial phosphates shown as spheres.

Two replicas of a 300 ns MD simulation of a ternary complex composed of PCNA, two p15 peptides spanning residues 47–70 and a 40 bp DNA (Supplementary Table ST1) were performed. The initial MD model was built by combining features of two different crystal structures: the p15^50–77^–PCNA complex (4) and the PCNA–dsDNA complex (5) by extending the DNA segment by 15 bp in each direction of the helical axis. The p15 peptide for this model was designed based on the p15^50–77^–PCNA complex structure, by deleting the seven p15 C-terminal residues that are flexible and do not interact with PCNA and adding three extra residues at the flexible N-terminus, as they are located inside the clamp channel, and may transiently interact with DNA. Before starting the simulation, the DNA was moved away from its binding site on the inner wall of the clamp channel to a central position with minimal contacts with PCNA. Along the trajectory, the p15 peptides stay anchored to their binding sites on two PCNA subunits, while DNA rotates and tilts towards the wall of the subunit that is not occupied by p15 (Figure 2A and B, Supplementary Movie S1 and Supplementary Figure S2). At the end of the simulation, the DNA segment within the channel has a position similar to that observed in a previous 250 ns simulation of PCNA bound to a 30 bp DNA in absence of p15, where DNA simultaneously interacts with two adjacent sets of DNA-helix matching residues located on two PCNA subunits, as well as with residues on the clamp back face (5) (Figure 2A). Importantly, the topology of polar interactions between DNA and the PCNA subunit not occupied by p15 coincides with that observed in the crystal structure of the PCNA–dsDNA complex (Figure 2C). These results are consistent with the crystal structure of the p15^50–77^–PCNA–DNA complex presented in this work and the proposed position for the partially disordered and/or incompletely occupied DNA. Altogether, these data suggest that, in the presence of two p15 molecules, a DNA duplex longer than 10 bp may still bind one of the three PCNA sliding surfaces.

Distance analysis of the intermolecular contacts along the MD trajectory shows that p15 residues N-terminal to the PIP-box (residues 52–61) are stably anchored to the inner wall of the PCNA ring, while the extreme N-termini (residues 47–51) remain flexible and thread the channel (Supplementary Figure S3 and Supplementary Movie S1). Overall, the p15 peptides establish limited contacts with DNA. In particular, polar contacts between peptide Y47 and G49 and DNA phosphates are detected. Conversely, DNA shows extensive interactions with the clamp (Figure 2A), and the side chains of many basic residues at the interface can randomly switch between adjacent DNA phosphates on a sub-nanosecond time scale (Supplementary Figure S4), as was observed in the MD simulation of the binary complex (5).

### NMR analysis of PCNA binding to the p15 PIP-box and a 10 bp DNA

**Figure 3.**
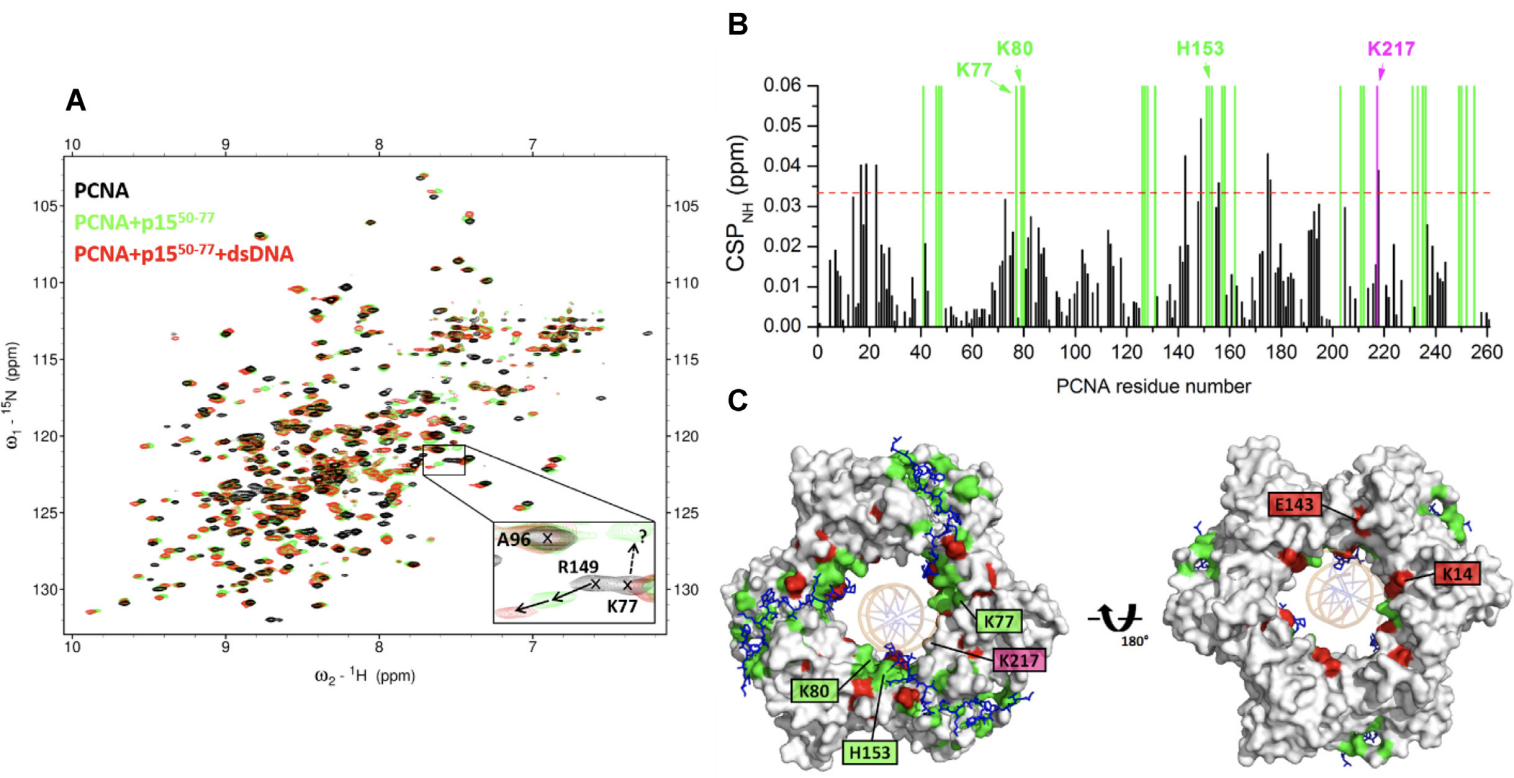
NMR analysis of PCNA binding to p15^50–77^ and a 10 bp dsDNA. (**A**) Superposition of ^1^H-^15^N TROSY spectra of 95 μM PCNA in the absence (black) and presence (green) of 606 μM of p15^50–77^ and (red) of 92 μM dsDNA (left) generated with oligonucleotides 3–4 in Supplementary Table S1. Spectra were acquired at 35°C on samples in 20 mM sodium phosphate, 50 mM NaCl, pH 7.0. The expansion shows signals of three representative residues. A96 signal is not perturbed by the addition of either p15^50–77^ or DNA. R149 signal persists upon p15^50–77^ addition, and shifts significantly by the sequential addition of DNA. Conversely, K77 signal disappears at substoichiometric concentrations of p15^50–77^, and is not recovered by DNA addition. The dotted arrow points to a signal that is tentatively assigned to K77 in the p15^50–77^-bound form. (**B**) Chemical shift perturbations (CSP) of backbone amide ^1^H and ^15^N NMR resonances induced by DNA. The dotted line indicates the average plus two standard deviations. The green bars indicate the position of residues that disappear upon addition of substoichiometric p15^50–77^, and are not drawn to scale. The residues perturbed by p15^50–77^ and that also appear at the interface of the p15^50–77^–PCNA–DNA crystal structure are labeled. (**C**) Front- and back-face views of PCNA surface. PCNA residues whose amide signals disappear in the presence of substoichiometric p15^50–77^, or are significantly perturbed by DNA are colored green or red, respectively. p15^50–77^ at the three PCNA PIP-box sites is shown in sticks, and DNA in the crystallographic position is shown as and orange ribbon.

We characterized the interaction of PCNA with p15^50–77^ and a 10 bp dsDNA (Supplementary Table ST1) by solution NMR. ^2^H–^15^N-labeled PCNA was firstly titrated with unlabeled p15^50–77^ and chemical shift perturbations of PCNA backbone amide signals analyzed. Two groups of interacting residues were identified: residues whose signals gradually shift along the titration (Figure 3A and Supplementary Figure S5), implying a fast exchange regime on the NMR time scale, and residues whose signals broaden and disappear (due to signal attenuation below the noise level or untraceable shifting) at substoichiometric concentrations of peptide (Figure 3A and B), indicating an intermediate exchange regime. For the residues of the first group, a dissociation constant of ∼35 μM at 35°C was derived (Supplementary Figure S6), at the same order of magnitude as the 12.5 μM constant previously measured at the same temperature by isothermal calorimetry (4). For some residues of the latter group, new signals appearing at saturation could be tentatively assigned to the bound form (Figure 3A). When projected onto the PCNA surface (Figure 3C), the residues whose signals disappear at substoichiometric peptide concentration strongly overlap with those at the interface in the p15^50–77^–PCNA–DNA crystal structure (Figure 1C), indicating that they interact tightly with PCNA, while the residues whose persisting signals significantly shift are located next to the main binding site (Supplementary Figure S5). Signals of K77, K80 and H153 disappear, while signal of K217 persists but is significantly perturbed (Figure 3B, C and Supplementary Figure S5). These are four of the five residues at the PCNA–DNA interface in the p15^50–77^–PCNA–DNA crystal structure (Figure 1B). This is consistent with the partial overlap between the p15 and DNA binding sites seen in the crystal structure, and suggests that, in solution, p15 may compete with DNA binding.

In order to map the interaction site of DNA onto p15-bound PCNA, a labeled PCNA sample saturated with unlabeled p15^50–77^ was used for a second titration with dsDNA. Signal shift saturation with dsDNA was achieved at 1:3 molar ratio (PCNA trimer:DNA duplex) (Figure 3A). The fact that, in an analogous titration of PCNA with dsDNA in the absence of p15^50–77^, only ∼10% of complex was formed at this molar ratio (5) indicates that the presence of p15^50–77^ increases the apparent DNA affinity for PCNA. This result is consistent with the fact that no DNA binding affinity can be biochemically measured for PCNA alone, whereas a weak but detectable affinity for DNA has been previously measured (4) by fluorescence polarization in the presence of p15^50–77^. Like in PCNA alone titrated with dsDNA or pDNA, backbone amide signal shifts are small (CSP < 0.06 ppm), suggesting that the interaction involves amino acids with long side chains (Figure 3B). The DNA-induced perturbations map to residues within the channel as well as residues that line the p15^50–77^ binding site on the front face of the PCNA ring (Figure 3C). The peptide, however, remains anchored to its binding site since the signals of PCNA residues that disappear in the presence of substoichiometric p15^50–77^ are not recovered by DNA addition (Figure 3A and B). Overall, these data suggest that DNA may thread through the PCNA channel when p15^50–77^ saturates the three PIP-box sites, but that p15^50–77^ remains anchored to the inner wall of the ring. The perturbations near the front face of the ring may arise from transient contacts that the threaded DNA makes with p15^50–77^, slightly altering the position of the side chains. Based on this data, however, we cannot discard the possibility that DNA only partially penetrates the PCNA channel saturated with p15^50–77^, either from the front or back face.

### p15-induced inhibition of pol η–PCNA holoenzyme activity

**Figure 4.**
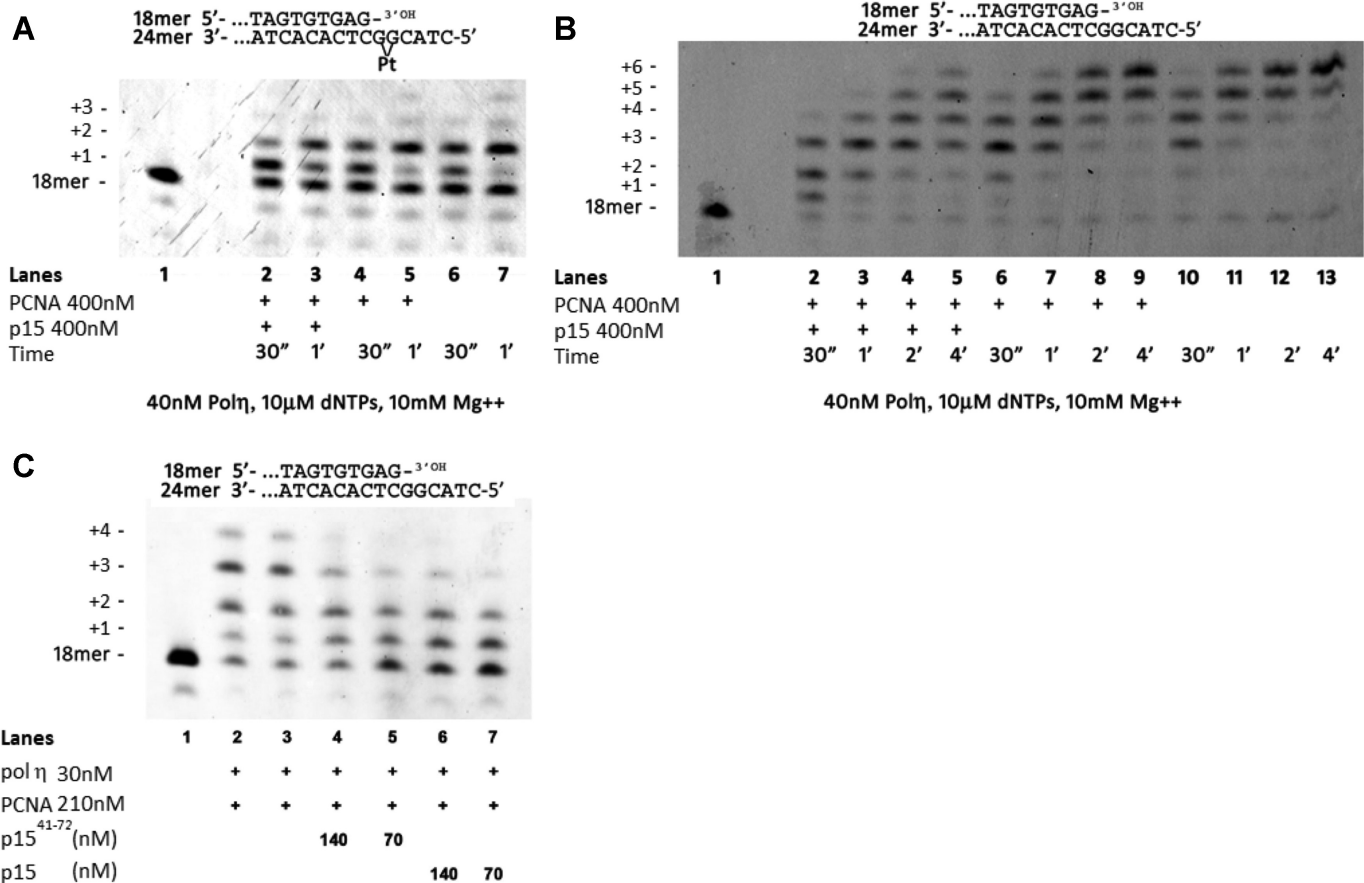
Inhibition of pol η holoenzyme by p15 (**A**) Time course of the reaction of pol η in the presence of PCNA/p15 at equimolar concentrations (Lanes 2 and 3), in the presence of PCNA (Lanes 4 and 5), or with pol η alone (lanes 6 and 7) on a cisPt(GG) template (10 nM), with all four dNTPs at the indicated concentration. (**B**) Time course of the reaction of pol η on the template without the lesion (10 nM), in the presence of PCNA/p15 at equimolar concentrations (lanes 2–5), in the presence of PCNA (lanes 6–9), or pol η alone (lanes 10–13), with all four dNTPs at the indicated concentration. (**C**) Reaction of pol η replicating the undamaged template in the presence of PCNA and in the absence or presence of p15^41–72^ peptide or full length p15. Reactants at the indicated concentrations were incubated at 37°C for 30 s and the reaction was stopped by addition of standard denaturing gel loading buffer. In all these experiments, PCNA was not ubiquitylated. These experiments show that p15 downregulates the activity of pol η–PCNA holoenzyme in bypassing a cisplatin lesion as well as in replicating a normal DNA substrate.

Given the importance of p15 in regulating the activity of pol η during TLS shown in cell-based experiments (18), we performed biochemical studies using purified proteins to gain further mechanistic insight in light of our new structural data. Thus, we first probed the effects of p15 on the activity of pol η–PCNA holoenzyme in extending a DNA primer across a site-specific cisplatin lesion. A time course of pol η bypass in the presence or absence of PCNA and p15 was performed with a DNA template bearing a cisPt(GG) adduct at positions +1 and +2 (Figure 4A). Data shows that pol η alone (40 nM) was able to complete the bypass, resulting in the incorporation of two dCMPs opposite both Gs in the adduct (lanes 6–7, Figure 4A). At increasing times, the +1 product was reduced, being converted into +2 product. Further elongation past the lesion was minimal, as expected from the highly distributive nature of pol η, especially in replicating damaged templates (36). Addition of a 10-fold excess of PCNA (lanes 4–5) did not significantly affect pol η activity. Such effect is not surprising: while PCNA has been shown to stimulate pol η activity on DNA substrates with blocked ends (36,37) or circular templates (38), this stimulation may not be captured on a DNA substrate with free ends as the one in our assay, because of the rapid turnover of PCNA across the substrate. In addition, if PCNA stimulation of pol η results from an increased affinity for the incorporated nucleotide (38), the saturating nucleotide concentrations used in our experiment would mask the stimulation. On the other hand, addition of equimolar amounts of PCNA and p15 (lanes 2–3) reduced nucleotide incorporation at +2 and +3 positions reproducibly also in independent experiments (Supplementary Figure S7). To rule out the possibility that the inhibitory effect of p15 on pol η activity is due to a defective loading of PCNA onto the primer-template (P/T) junction of the DNA substrate, the TLS experiment was repeated in the presence of *Saccharomyces Cerevisiae* Replication Factor C (ScRF-C), which is able to load human PCNA on DNA (39). Our results (Supplementary Figure S8) show that p15 delays TLS by pol η–PCNA across a cis-Pt lesion even in the presence of RF-C.

The replication experiment was repeated with the same DNA substrate without the lesion (Figure 4B). Again, while pol η alone (lanes 10–13) or in the presence of PCNA (lanes 6–9) showed equal processivity, the addition of PCNA and p15 reduced nucleotide incorporation at position +2 at the initial time point (lane 2), and slowed down the synthesis of the full-length product (lanes 3–5). To confirm that the inhibitory effect of p15 is mediated by the interaction with PCNA and not by a direct interaction with pol η, the activity of pol η in replicating the substrate was tested in the absence or presence of p15 alone, showing that p15 alone does not affect the activity of the polymerase (Supplementary Figure S9). To assess whether a p15 fragment spanning the region interacting with PCNA is sufficient to induce the inhibitory effect on pol η observed with full length p15, pol η activity was tested on the undamaged DNA substrate (Figure 4C) in the presence of PCNA and in the absence (lanes 2 and 3) or in the presence of p15^41–72^ peptide (lanes 4,5) or full length p15 (lanes 6,7). Addition of p15^41–72^ or p15 in combination with PCNA caused a reduction of DNA synthesis with respect to pol η and PCNA alone, which stopped at positions +1 and +2, corresponding to incorporation opposite the first two Gs of the template. These results indicate that p15 or p15^41–72^ in conjunction with PCNA are able to reduce pol η synthesis at comparable levels.

Considering the concentrations of pol η, PCNA and p15 in the assays in Figure 4A and B, and the dissociation constants for the pol η–PCNA (*K*_d_ = 0.4 μM, measured by surface plasmon resonance at 25°C) (40) and p15–PCNA (*K*_d_ = 1.1 μM, measured by ITC at 25°C) (4) binding equilibria, we estimated the relative populations of binary complexes assuming that binding of pol η and p15 to PCNA is mutually exclusive. Under this assumption, 42% of pol η and 23% of p15 are saturated with PCNA. Thus, each dNTP insertion step is carried out by a combination of pol η alone and pol η holoenzyme. However, if p15 did inhibit binding of pol η to PCNA, a drop of pol η activity in the presence of p15 should not be observed, because the latter would favor the formation of free pol η, which shows full activity. Furthermore, the relatively high (30 nM) (36) and low (5 μM) (4) affinity of pol η and p15 for DNA, respectively, rules out that p15 may prevent the access of pol η to the DNA P/T junction. This suggests that inhibition of pol η processivity in the presence of PCNA and p15 is due to the formation of an impaired ternary p15–PCNA–pol η holoenzyme, deficient in primer synthesis against both damaged and normal templates.

## DISCUSSION

### Topology and stoichiometry of p15 binding to PCNA loaded on DNA

**Figure 5.**
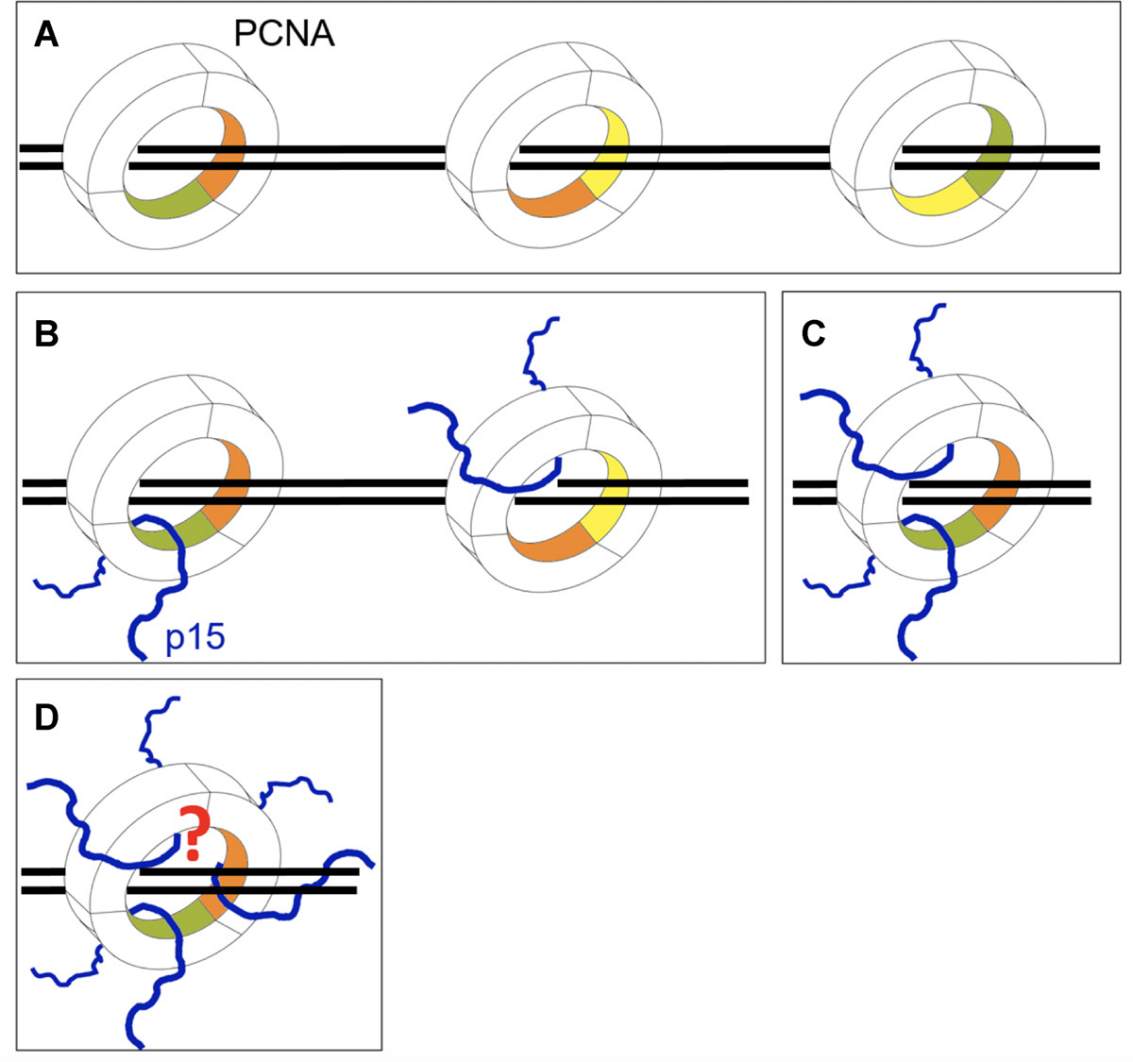
Possible effects of p15 on PCNA sliding (**A**) PCNA can diffuse on DNA contacting three equivalent sliding surfaces, each composed of two homologous sets of basic residues spanning across the interface of two subunits (the 3 PCNA subunits are colored green, yellow and wheat). (**B–D**) The stoichiometry of p15 binding to the PCNA homotrimer defines the available surfaces for clamp sliding. Whether a configuration where PCNA simultaneously binds three p15 chains and DNA, can be achieved, and whether it completely or partially hinders sliding, remains to be determined.

The data reported in this study reveals that DNA can thread through the PCNA ring along with two p15 chains, and that the structure of the p15 segment interacting with PCNA is invariant in the absence or presence of DNA. In our MD simulations, the disordered N-termini of the p15 fragments exit the PCNA back face, a topology analogous to that of full length p15 bound to PCNA in the absence of DNA (4,21). The limited number of contacts between p15^50–77^ and DNA observed in our computational analysis suggests that p15 mostly operates as a passive steric obstacle constraining the DNA in the clamp channel. Critically, p15 shields key residues at the PCNA sliding surfaces, confining DNA in discrete positions, which depend on the p15 stoichiometry of binding (Figure 5A–D). This mode of binding, with DNA partially competing with p15 for a single binding site on the clamp inner ring, is also supported by (i) our NMR study showing that DNA does not disengage p15 from the inner wall of PCNA saturated with p15 and (ii) our structure of PCNA co-crystallized with three p15 peptides and DNA, showing that DNA does not occupy the central channel.

Because p15 is stably associated with PCNA on chromatin during the S phase of the cell cycle (18), it is likely that during replication polymerases and other DNA-editing enzymes bind one or two PCNA sites only, so that the free sites(s) would be available for p15 binding. Indeed, the catalytic subunit of pol δ (p125) and Fen-1 were co-precipitated with p15 and PCNA from pancreatic cell lysates (19). While Fen-1 is a monomeric enzyme that binds PCNA through a single PIP-box (41), human pol δ consists of four subunits (p125, p66, p50 and p12) and all of them are required for optimal holoenzyme activity (42). Although all four subunits contain potential PIP-box sites, examination of reconstituted holoenzymes in which the PCNA binding motifs have been mutated or inactivated have only been tested for p12 and p66 (43–45), and there may exist multiple subassemblies of pol δ *in vivo* (46,47). Thus, considering its small size and high flexibility, p15 may coexist with pol δ on the same PCNA homotrimer in a replicating cell. Likewise, a comparison of the Fen-1–PCNA (41) (PDB ID: 1UL1) and p15^50–77^–PCNA (4) (PDB ID: 4D20) crystal structures suggests that Fen-1 and p15 may both be accommodated on a single PCNA ring.

**Figure 6.**
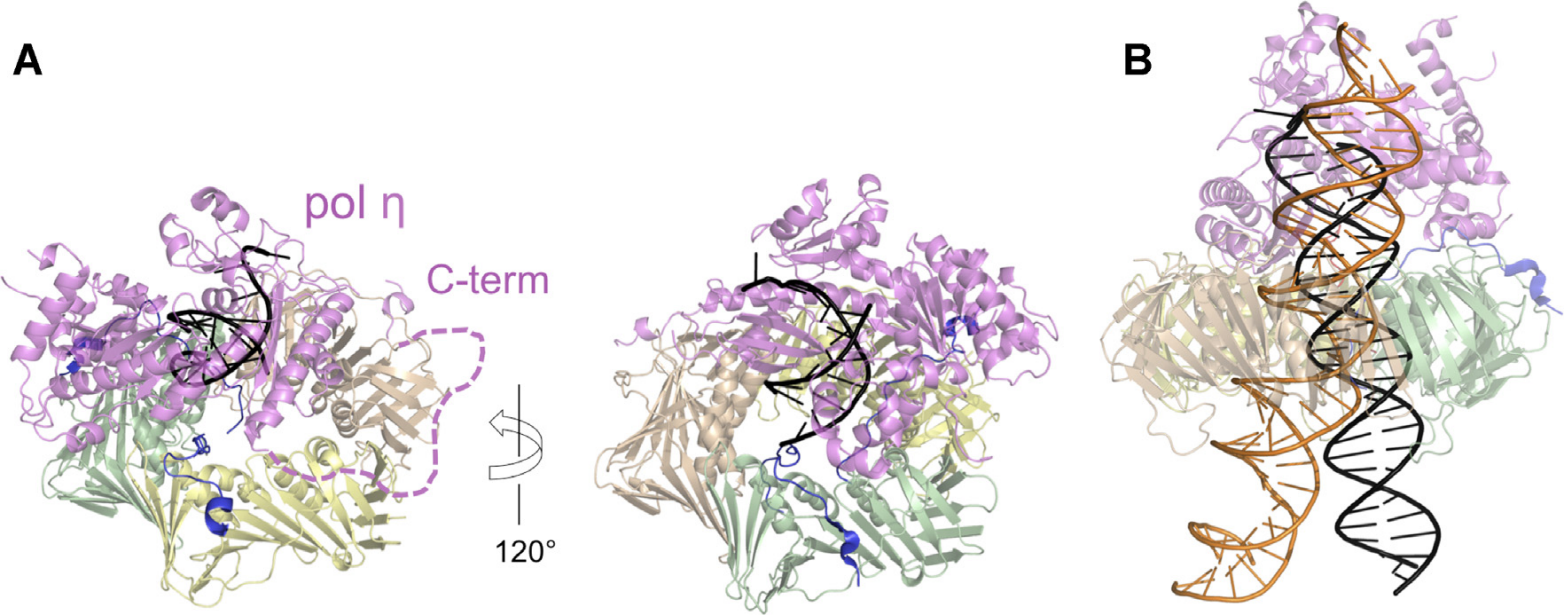
Structural models of pol η-PCNA holoenzymes with p15 and DNA. (**A**) The PCNA trimer of the p15^50–77^–PCNA crystal structure (PDB ID: 4D2G) was superposed to PCNA of the low-resolution structure of human pol η–PCNA–DNA generated from EM data (PDB ID: 3JA9 and 3JAA) (33). DNA is shown in black. The vacant PIP-box site on PCNA (subunit wheat) was occupied by the C-terminal PIP-box of pol η using the crystal structure of human PCNA bound to pol η residues 700–710 (chain W of PDB ID 2ZVK) (40). The dashed line indicates the flexible pol η C-terminus (residues 433–699). (**B**) The PCNA trimer of the structure of p15^50–77^–PCNA–DNA complex (PDB: 6EHT) was superposed to PCNA of the pol η–PCNA–DNA (48) complex (PDB: 3JA9 and and 3JAA). The DNA of the first complex (elongated to 40 bp) is shown as an orange ribbon, that of the latter (elongated to 25 bp) as a black ribbon. According to these models, it is possible that p15 may co-exist with pol η on the same PCNA ring. However, the constraint on DNA within the clamp channel imposed by p15 may hinder the translocation of pol η holoenzyme on DNA.

The 78-kDa pol η is less bulky than pol δ and binds PCNA mainly through a single PIP-box located at its flexible C-terminus (40). Considering the comparable PIP-box affinities for PCNA (4,40), pol η and p15 may co-exist on one PCNA homotrimer. The recently determined negative stain EM structure of ubiquitylated PCNA bound to pol η and DNA (48), where two PIP-box sites would be free for p15 binding, supports this hypothesis (Figure 6A).

### Possible roles of p15 in the holoenzymes with the replicative and TLS polymerases

We propose that p15 is part of the human holoenzyme that replicates the DNA lagging strand, and may function to fasten the DNA within the clamp channel by reducing the accessible sliding surfaces. We and others have shown that the PCNA–DNA interaction is weak and transient (2,4,5,7,9), and recent evidence showed that human pol δ maintains a loose association with PCNA while replicating (49). Because the polymerase needs to maintain a fixed position relative to the phosphodiester backbone at the P/T junction, a constrained orientation of PCNA with respect to the helical pitch may improve the overall stability of the holoenzyme. Such stabilizing role of p15 would explain the negative effects of p15 knockdown on DNA synthesis (19,20).

While a high-resolution structure of a PCNA–polymerase–DNA complex is still awaiting, both the medium resolution EM structure (50) and MD simulations of *Pyrococcus furiosus* (Pfu) PCNA bound to PolB and DNA (51) show that DNA within the clamp is tilted. Particularly, the MD model of the complex in polymerizing mode shows features at the clamp–DNA interface analogous to those observed in the p15^50–77^–PCNA–DNA model presented here, where five conserved positively charged residues, matching the dsDNA B-helix architecture, interact with five consecutive phosphates of one DNA strand, suggesting that this key determinant of the interaction is conserved and may be present in the pol δ holoenzyme. Indeed, mutation of residues at the PCNA–DNA interface impairs both initiation of DNA synthesis (10) and processivity of pol δ (11), suggesting that the PCNA–DNA interactions control both clamping and sliding activities of PCNA in processive DNA replication. In their computational work, Ivanov and co-workers (51) also showed that the repositioning of the PolB core during the conformational switch from polymerizing to editing modes forces the DNA to tilt from one side of the PfuPCNA channel to the other. A 30° change in DNA tilt within the clamp in the catalytic core of the bacterial replisome from polymerizing to editing modes was also observed in the recent cryo-EM work by Lamers and colleagues (52,53). Perhaps, in the human pol δ holoenzyme, p15 plays a role in guiding DNA through the PCNA inner rim in between DNA synthesis and editing steps of the polymerase.

Single molecule experiments suggested that PCNA may slide by rotationally tracking the DNA helix or by a less frequent translational mode uncoupled from the helical pitch (8), while a recent computational work predicts that the coupling between rotation and translation is weak (9), suggesting that the translational mode is prevalent. Thus, p15 binding to PCNA may increase the rotation-translation coupling by reducing the available sliding surfaces (Figure 5). This, together with the DNA binding activity of the p15 disordered N-terminus, may result in a slower diffusion of PCNA on DNA. Therefore, p15 might regulate the sliding velocity of PCNA, and this function may be required for the DNA damage response to prevent a rapid drift of PCNA from stalled forks in between polymerase swapping events.

Upon encounter of a DNA lesion, pol δ dissociates from PCNA, which becomes ubiquitylated, and is replaced by pol η that replicates DNA past the lesion (54,55). While the ubiquitin moieties of ubiquitylated-PCNA may interact with the C-terminal ubiquitin binding motif (UBZ) of pol η, a large body of data argues that ubiquitylation of PCNA is not strictly necessary for pol η recruitment and activity in TLS (38,56,57). Notably, a recent report unambiguously demonstrates that the binding of pol η to PCNA, and DNA synthesis by a pol η holoenzyme are both independent of PCNA monoubiquitylation (36).

During unperturbed replication, p15 is mono-ubiquitylated at K15 and K24 and is degraded by the proteasome after UV irradiation or cisplatin treatment (18). Degradation of ubiquitylated p15 upon DNA damage is required for the recruitment of pol η to the replication foci and efficient lesion bypass, and the authors suggested that p15 may prevent the binding of pol η to PCNA. In this work, we showed that p15 has an inhibitory activity on the pol η–PCNA holoenzyme in synthesizing past the 5′dG of a 1,2-d(GpG) cisplatin DNA adduct and in extending the undamaged template. Importantly, a p15 fragment spanning only the region of interaction with PCNA is sufficient to inhibit pol η activity. Rather than preventing pol η from binding to PCNA, our data suggests that p15 inhibits pol η activity by associating to the holoenzyme, a possibility supported by structural considerations (Figure 6A). Although the DNA in the EM map of pol η bound to ubiquitylated PCNA and DNA is not fully defined (48), the DNA duplex within PCNA lies close to one of the clamp subunits, in a position different from that observed in our p15–PCNA–DNA complex structure (Figure 6B). We propose that the constraint imposed to DNA by p15 in the central channel of PCNA may hinder the advancement of DNA in the pol η active site required for the incorporation of the nucleotide opposite to the 5′dG of the DNA template. Thus, after the insertion of the first dCMP, the polymerase may become ‘idle’ and dissociate from PCNA. Further high-resolution structural studies on pol η holoenzyme will shed light on this possibility.

## DATA AVAILABILITY

Atomic coordinates of p15^50–77^–PCNA–DNA and p15^41–72^–PCNA complexes have been deposited in the Protein Databank under the accession codes 6EHT and 6GWS, respectively. Assignments of backbone amide NMR resonances of human PCNA bound to p15^50–77^ and DNA are deposited in the Biological Magnetic Resonance database BMRB under accession code 27558.

## Supplementary Material

Supplementary Data
