# Supplementary material for: p15PAF binding to PCNA modulates the DNA sliding surface
Source: Nucleic Acids Res. 2018 Aug 8;46(18):9816–28. doi: 10.1093/nar/gky723 (PMC6182140; doi:10.1093/nar/gky723)
Supplement: Supplementary Data [file gky723_supplemental_files.zip › SI_NAR_acc.pdf]

Supplementary Information for

p15^{PAF} binding to PCNA modulates the DNA sliding surface

Matteo De March[1], Susana Barrera-Vilarmau[2], Emmanuele Crespan[3], Elisa Mentegari[3], Nekane Merino[4], Amaia Gonzalez-Magaña[4], Miguel Romano-Moreno[4], Giovanni Maga[3], Ramon Crehuet[2], Silvia Onesti[1], Francisco J. Blanco[4,5] and Alfredo De Biasio[1,6]

[1] Structural Biology Laboratory, Elettra-Sincrotrone Trieste S.C.p.A., Trieste 34149, Italy;

[2] Institute of Advanced Chemistry of Catalonia (IQAC), CSIC, Jordi Girona 18-26, 08034, Barcelona, Spain;

[3] Institute of Molecular Genetics, IGM-CNR, via Abbiategrasso 207, 27100 Pavia, Italy

[4] CIC bioGUNE, Parque Tecnològico de Bizkaia Edificio 800, 48160 Derio, Spain;

[5] IKERBASQUE, Basque Foundation for Science, Bilbao, Spain

[6] Leicester Institute of Structural & Chemical Biology and Department of Molecular & Cell Biology, University of Leicester, Lancaster Rd, Leicester LE1 7HB, UK

This file includes:

Supplementary Figures S1-9

Supplementary Tables T1-2

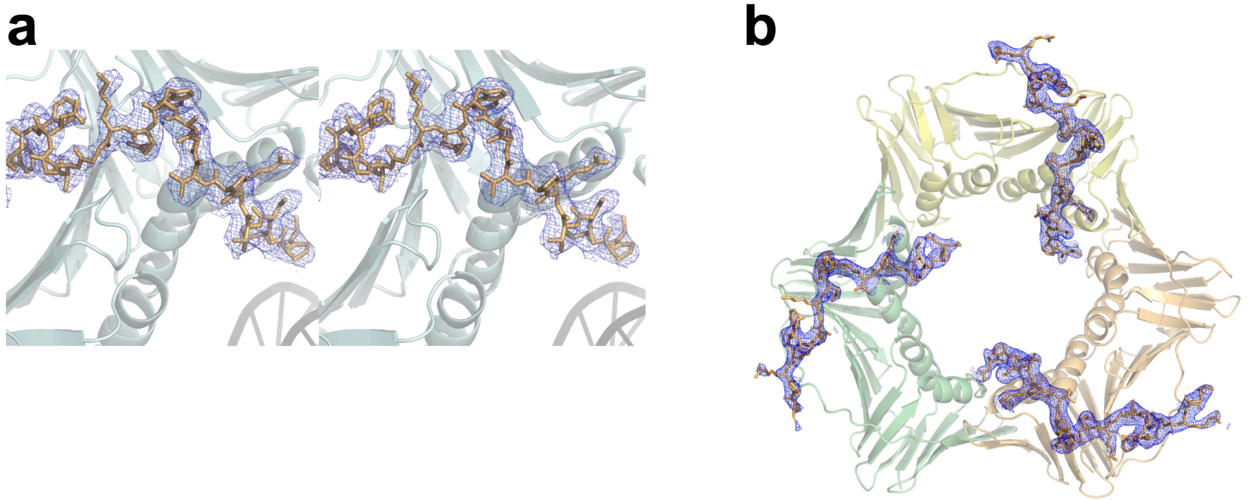

Supplementary Figure S1. (**a**) Stereo view of the $2F_o$-F_c electron density map around the p15$^{50-77}$ peptide with higher occupancy in the p15$^{50-77}$–PCNA–DNA complex, contoured at 1 σ (**b**) View of the $2F_o$-F_c map of the p15$^{41-72}$–PCNA complex, contoured at 1 σ around the three p15$^{41-72}$ peptides. In both panels, p15 peptides are in stick representation, PCNA in ribbon representation.

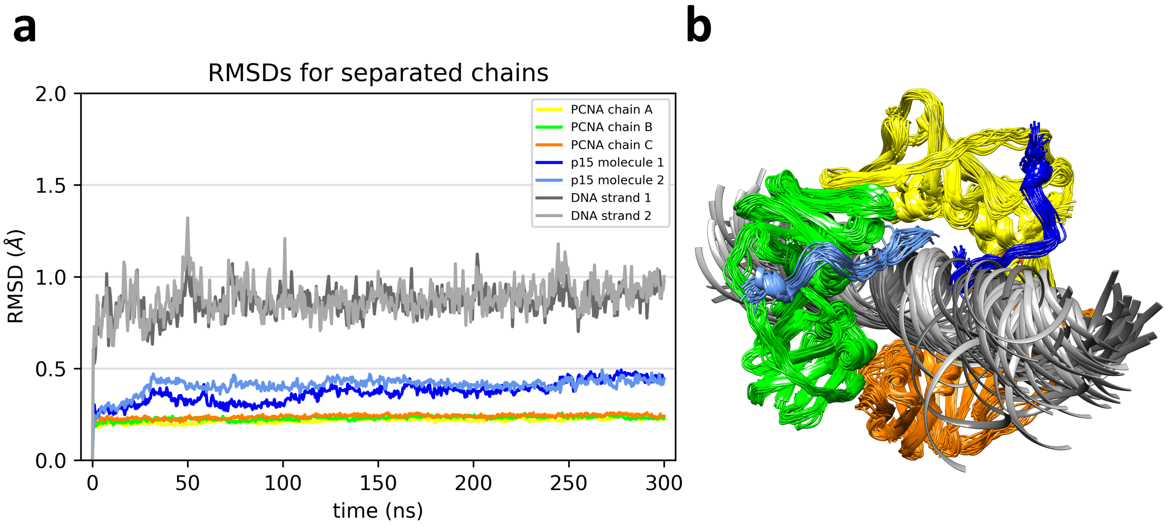

Supplementary Figure S2. (a) Root Mean Square Deviation (RMSD) calculated for the backbone heavy atoms of the PCNA protein, the DNA duplexes, and p15 peptides along the MD trajectories. In each case, the system has been superimposed onto the initial minimized structure that was built from the crystal structure, as explained in the methods section. (**b**) Superposition of the ternary complex structures from MD replica 1, with colour code as in (**a**). One can see that most of DNA flexibility arises from its terminal regions. The results for replica 2 are visually similar.

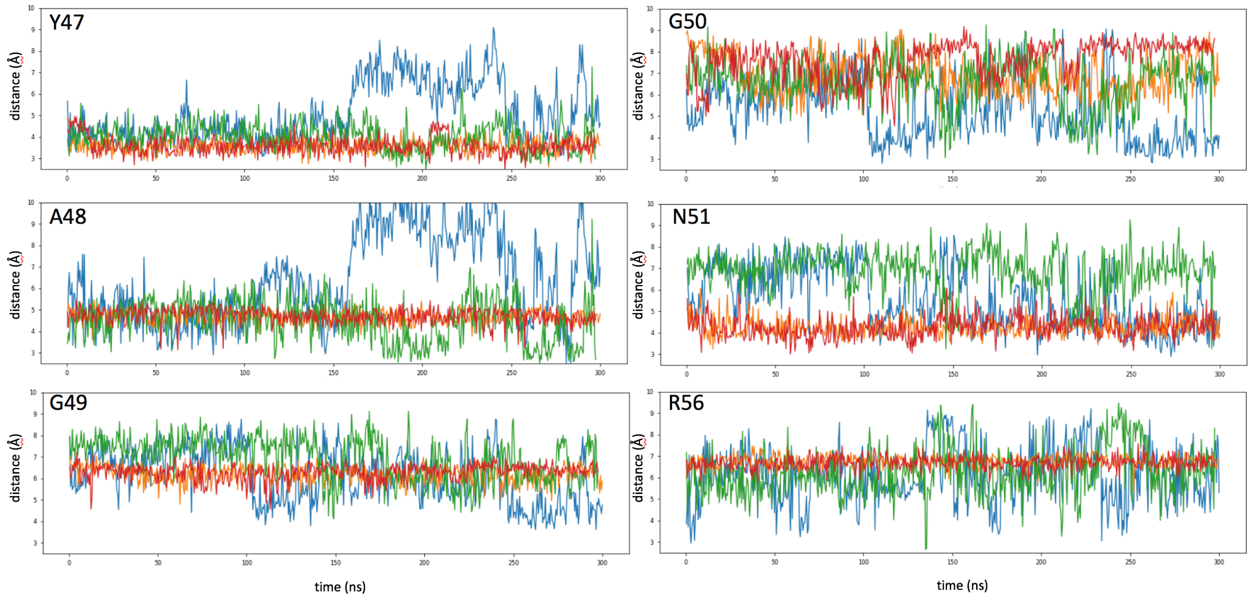

Supplementary Figure S3. *Analysis of the interaction of the p15 with DNA in the MD simulation of PCNA bound to two p15$^{47\text{-}70}$ peptides and a 40 bp DNA duplex*. Evolution of distances between p15$^{47\text{-}70}$ peptide residues (backbone or side-chain atoms) to DNA phosphorus (P) in the MD trajectory for the indicated residues (red and orange traces correspond to peptide 1 in MD replica 1 and 2, respectively; blue and green traces correspond to peptide 2 in replica 1 and 2, respectively).

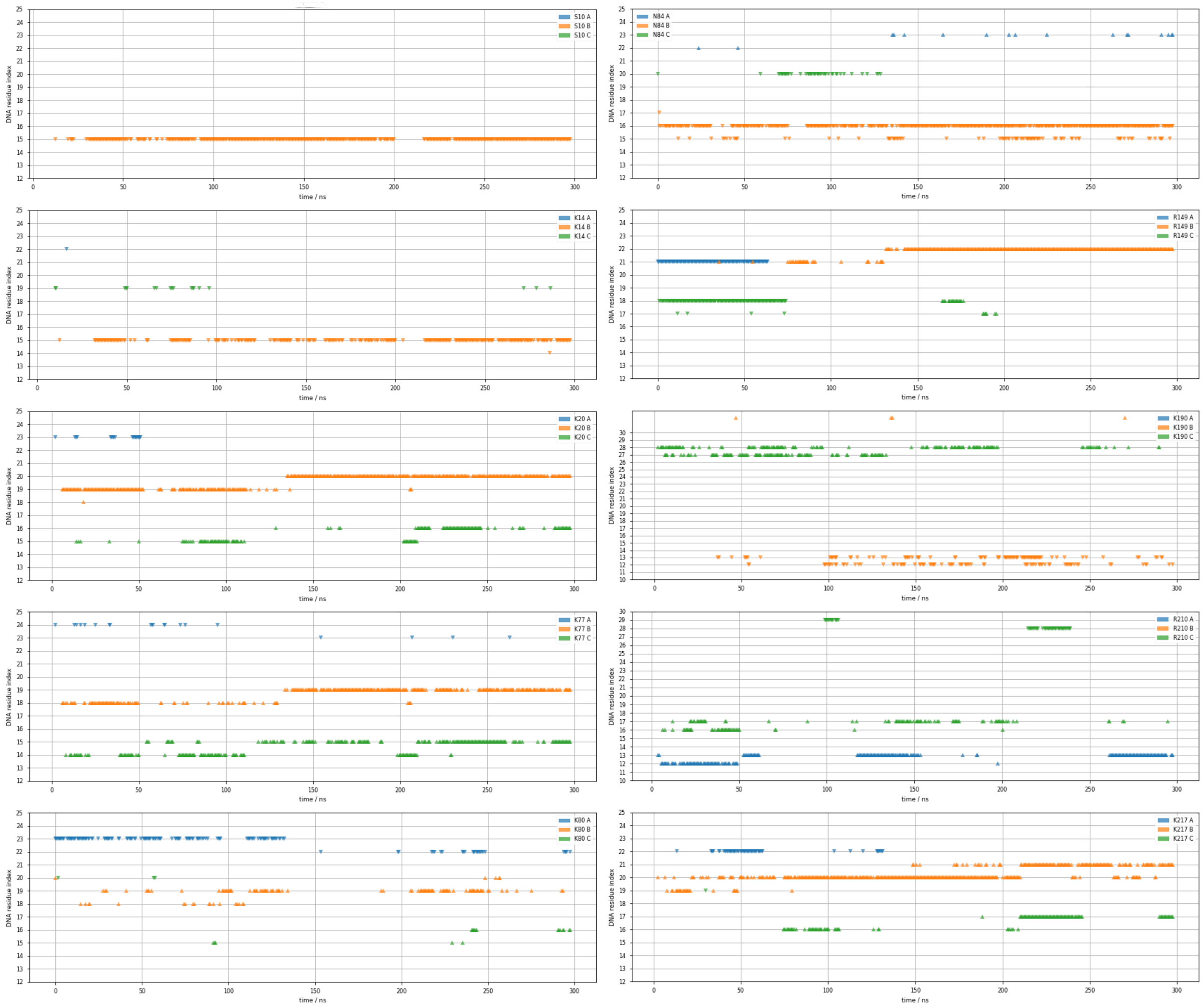

Supplementary Figure S4. *Analysis of the MD simulation of PCNA bound to two p15$^{47\text{-}70}$ peptides and a 40 bp DNA duplex*. Time evolution of contacts (interatomic side chain nitrogen or oxygen – DNA phosphorus distance < 4.7 Å) between PCNA interfacial residues and DNA phosphorus atoms. Contacts with residues belonging to PCNA subunit A, B and C are shown as triangles colored in blue, orange and green, respectively. The interacting nucleotides of the two strands of the 40 bp dsDNA are consecutively numbered, and triangles with vertexes pointing up and down correspond to contacts with DNA strand 1 and 2, respectively.

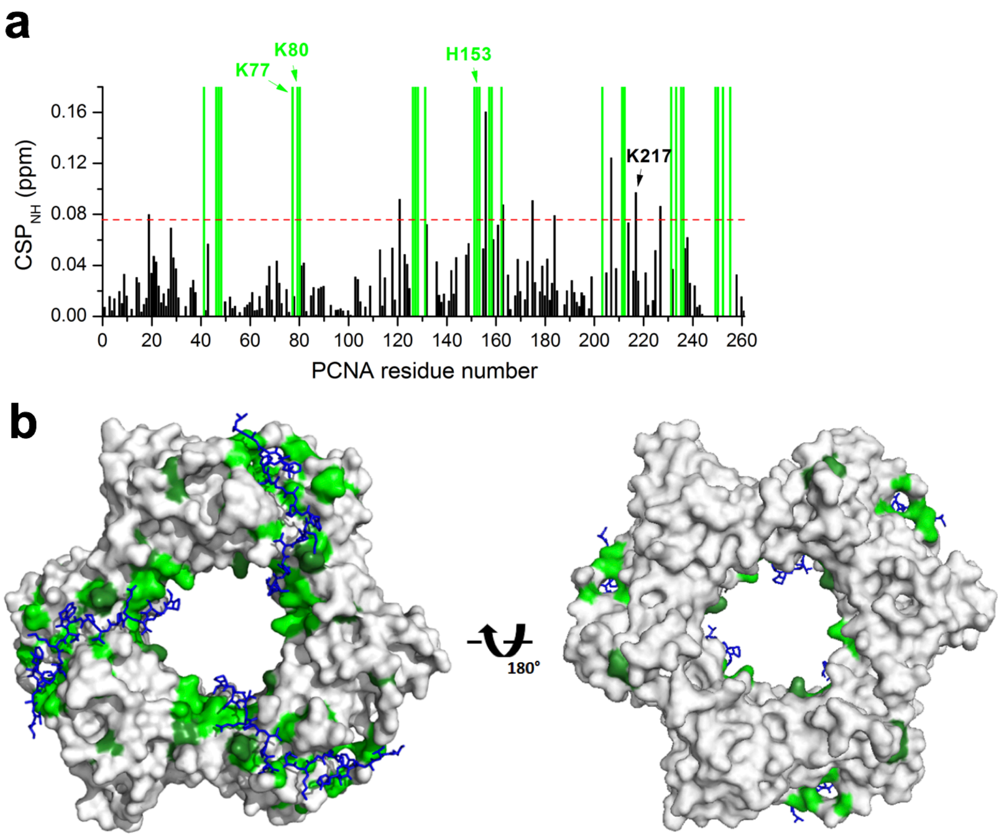

Supplementary Figure S5 (**a**) Chemical shift perturbations (CSP) of PCNA backbone amide ^{1}H and ^{15}N NMR resonances induced by p15$^{50\text{-}77}$. The dotted line indicates the average plus two standard deviations. The green bars indicate the position of residues that disappear upon addition of substoichiometric p15$^{50–77}$, and are not drawn to scale. The residues perturbed by p15$^{50–77}$ and that also appear at the PCNA–DNA interface in the p15$^{50–77}$–PCNA–DNA crystal structure are labeled (**b**) Front- and back-face views of PCNA surface. PCNA residues whose amide signals disappear in the presence of substoichiometric p15$^{50–77}$, or whose signals persist but shift significantly along the titration are colored light or dark green, respectively. p15$^{50–77}$ bound to the three PCNA PIP-box sites is shown in sticks.

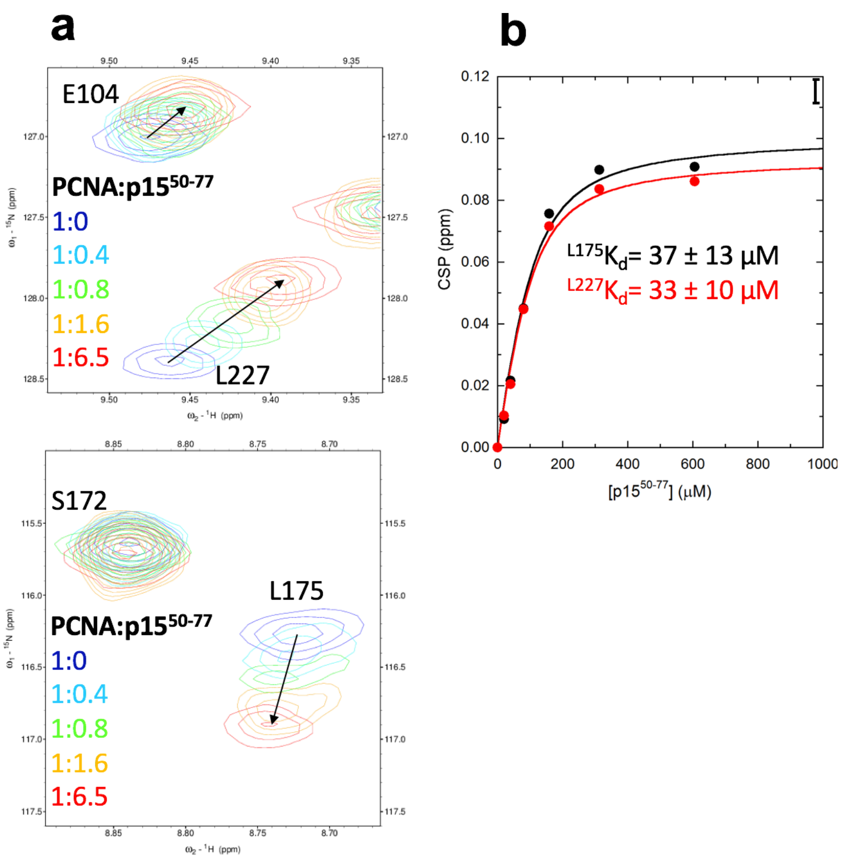

Supplementary Figure S6. *NMR titration analysis of PCNA binding to p15$^{50\text{-}77}$*. (**a**) Overlay of ^{1}H-^{15}N TROSY spectra of 100 µM PCNA in the presence of increasing concentrations of unlabeled p15$^{50\text{-}77}$, as indicated by the PCNA protomer : p15 peptide molar ratios. For the sake of clarity only five points along the titration are plotted. Spectra were acquired at 35 °C on samples in 20 mM sodium phosphate, 50 mM NaCl, pH 7.0. The selected region shows significantly perturbed residues (L175, L227) and two unperturbed residues (S172, E104). Arrows indicate the peak center of the residue at the last titration point. The steady shifts of the signals imply a fast exchange between free and bound PCNA with respect to the NMR chemical shift time scale (**b**) Analysis of NMR CSP of two PCNA residues at increasing concentrations of p15$^{50\text{-}77}$, using a single-site binding model. The errors in the K_D values are fitting errors. The CSP experimental error estimated from the digital resolution of the spectra is 0.009 ppm, which is the size of the bar at the right upper corner of the plot.

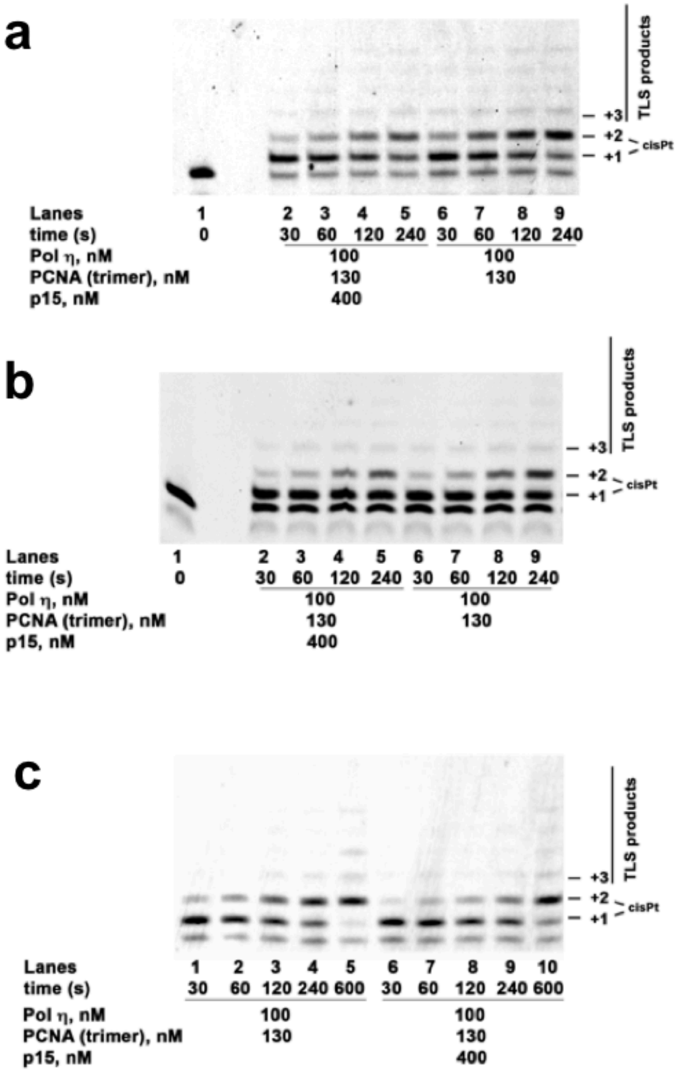

Supplementary Figure S7. (a-c) Three independent time course repeats of the reaction of pol η in the presence of PCNA/p15 at the indicated concentrations on the cisPt(GG) template (10nM).

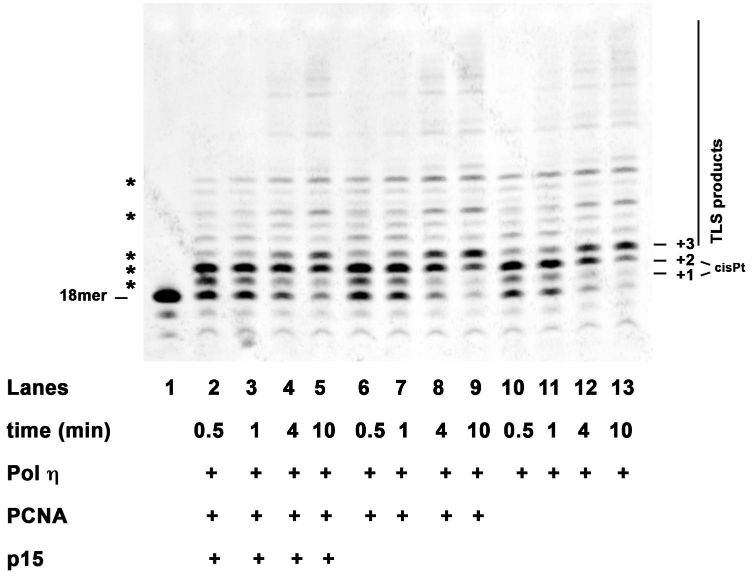

Supplementary Figure S8.

Inhibition of pol η activity by PCNA-p15 in the presence of RF-C. Pol η was tested on the 18/24mer primer-template substrate bearing a cis-Pt adduct at the two guanines at positions +1 and +2 of the template strand, in the presence of RF-C and in the absence (lanes 10-13) or in the presence of PCNA (lanes 6-9) or PCNA and p15 (lanes 2-5). Reactant concentrations were: 50 μM dNTPs, 0.5 mM ATP, 150 nM ScRF-C, 400 nM Pol η, 400 nM p15, 50 nM PCNA, 40 nM Cis-Pt 18/24mer. Time course of nucleotide incorporation showed that PCNA did not significantly change the translesion synthesis products with respect to pol η alone. However, addition of p15 in combination with PCNA, caused an accumulation of +1 and +2 products, corresponding to incorporation opposite the two G's of the cis-Pt adduct, and a delay in the appearance of longer products, particularly at short incubation times (compare lanes 2,3 with lanes 6,7 and 10, 11), as can be seen comparing the products marked with asterisks across the different lanes. Thus, these results indicated that the PCNA/p15 complex was able to delay TLS across a cis-Pt lesion even in the presence of RF-C.

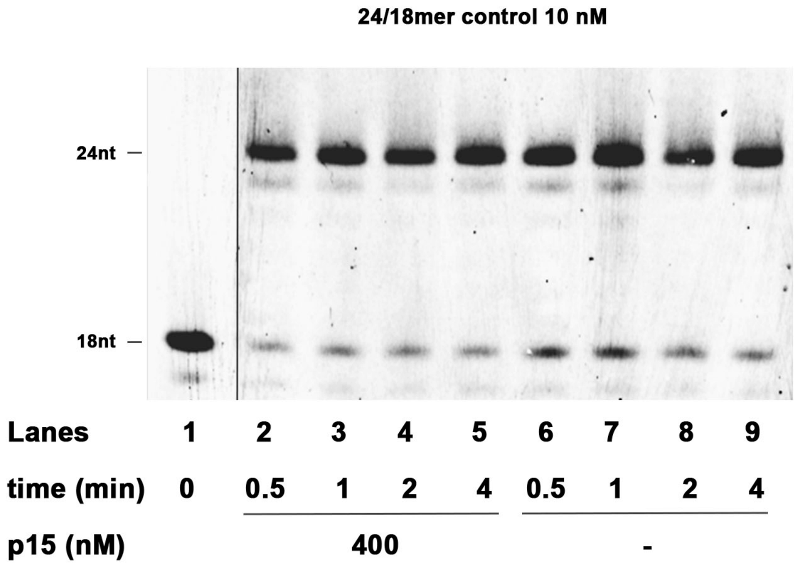

Supplementary Figure S9.

Time course of the reaction of pol η (70 nM) alone (Lanes 6-9) or in the presence of p15 (400 nM) (Lanes 2-5) on the 24/18mer control template (10 nM). Bar indicates were two portions of the same gel have been moved close for clarity.

Supplementary Table T1. Sequences of the oligonucleotides used in this study.

DNA oligo	Sequence (5´-3´)
1	Cy5-CCCATCGTAT
2	TTTTATACGATGGG
3	CCCATCGTAT
4	ATACGATGGG
5	ATACGATGGGATACGATGGGATACGATGGGATACGATGGG
6	CCCATCGTATCCCATCGTATCCCATCGTATCCCATCGTAT
7	CTAC**GG**CTCACACTATCTCACACT
8	CTACGGCTCACACTATCTCACACT
9	AGTGTGAGATAGTGTGAG

The sequence of oligonucleotides 1 and 2 were used to form the primed DNA and correspond to the substrate used in the crystal structure of β-clamp bound to pDNA, reported by Georgescu *et al.*[1]. Oligonucleotides 3 and 4 were used to form the dsDNA duplex for co-crystallization with PCNA and $p15^{50-77}$ and for NMR titration. Oligonucleotides 5 and 6 were used to form the 40 bp DNA for the MD simulation. Oligonucleotides 7-9 were used for the DNA synthesis studies with pol η. The bold letters in oligo 7 indicate the position of the cisPt(GG) crosslink. Oligo 8 represents the 24-mer undamaged template, and the sequence underlined is the one complementary to oligo 9 (18-mer primer).

[1] Georgescu, R.E., Kim, S.S., Yurieva, O., Kuriyan, J., Kong, X.P. and O'Donnell, M. (2008) Structure of a sliding clamp on DNA. *Cell*, **132**, 43-54.

Supplementary Table T2: Data collection and refinement statistics.

	PDB: 6EHT<br>p15$^{50\text{-}77}$–PCNA–pDNA	PDB: 6GWS<br>p15$^{41\text{-}72}$– PCNA
**Data collection**		
Space group	$P2_1$	$P2_1$
Cell dimensions		
a, *b*, *c* (Å)	75.99, 42.30, 141.83	79.24, 89.75, 85.13
α, β, γ (°)	90, 102.70, 90	90, 117.25, 90
Resolution (Å)	46.12 - 3.20 (3.37 - 3.20)	75.69 - 2.90 (3.08 - 2.90)
R_{merge}	0.105 (0.320)	0.11 (1.113)
R_{PIM}	0.087 (0.257)	0.087 (0.730)
$I / \sigma I$	4.5 (2.0)	4.77 (1.6)
CC(1/2)	0.987 (0.916)	0.986 (0.763)
Completeness (%)	95.3 (98.8)	92.2 (91.3)
Redundancy	2.2 (2.3)	1.8 (1.8)
**Refinement**		
Resolution (Å)	40.484 - 3.2	44.9 - 2.9
No. reflections	13440	23688
R_{work} / R_{free}	0.26 / 0.33	0.20 / 0.26
No. atoms		
Protein	5229	5882
Peptide	277	538
DNA	410	-
Water	9	10
B-factors (Å^2)		
Protein	60.6	99.04
Peptide	70.6	98.99
DNA	230.1	-
Water	60.5	70.65
R.m.s. deviations		
Bond lengths (Å)	0.0102	0.0030
Bond angles (°)	1.3961	1.2740
